# Single-cell multi-omics and machine learning for dissecting stemness in cancer

**DOI:** 10.1093/bib/bbaf566

**Published:** 2025-10-29

**Authors:** Xinyang Huang, Shenghui Huang, Chiara Reina, Berina Šabanović, Miriam Roberto, Alexandra Aicher, Jiajia Tang, Christopher Heeschen

**Affiliations:** Center for Single-Cell Omics, School of Public Health, Shanghai Jiao Tong University School of Medicine, 227 South Chongqing Road, Huangpu District, 200025 Shanghai, China; State Key Laboratory of Systems Medicine for Cancer, Shanghai Jiao Tong University School of Medicine, 227 South Chongqing Road, Huangpu District, 200025 Shanghai, China; Department of Molecular Biotechnology and Health Sciences, University of Turin, Via Nizza 52, 10126 Turin (Torino), Italy; Pancreatic Cancer Heterogeneity, Candiolo Cancer Institute - FPO - IRCCS, Strada Provinciale 142 Km 3,95, 10060 Candiolo (Torino), Italy; Pancreatic Cancer Heterogeneity, Candiolo Cancer Institute - FPO - IRCCS, Strada Provinciale 142 Km 3,95, 10060 Candiolo (Torino), Italy; Pancreatic Cancer Heterogeneity, Candiolo Cancer Institute - FPO - IRCCS, Strada Provinciale 142 Km 3,95, 10060 Candiolo (Torino), Italy; Pancreatic Cancer Heterogeneity, Candiolo Cancer Institute - FPO - IRCCS, Strada Provinciale 142 Km 3,95, 10060 Candiolo (Torino), Italy; Precision Immunotherapy, Graduate Institute of Biomedical Sciences, China Medical University, No. 91, Xueshi Road, North District, Taichung City 404328, Taiwan, Taiwan (R.O.C.); Center for Single-Cell Omics, School of Public Health, Shanghai Jiao Tong University School of Medicine, 227 South Chongqing Road, Huangpu District, 200025 Shanghai, China; State Key Laboratory of Systems Medicine for Cancer, Shanghai Jiao Tong University School of Medicine, 227 South Chongqing Road, Huangpu District, 200025 Shanghai, China; Pancreatic Cancer Heterogeneity, Candiolo Cancer Institute - FPO - IRCCS, Strada Provinciale 142 Km 3,95, 10060 Candiolo (Torino), Italy

**Keywords:** cancer stem cells, cellular plasticity, therapy resistance, tumor heterogeneity, single-cell omics, artificial intelligence (AI), machine learning (ML), deep learning (DL)

## Abstract

Cancer stem cells (CSCs) are a subpopulation of tumor cells with self-renewal capacity and the ability to drive tumor growth, metastasis, and relapse. They are widely recognized as major contributors to therapeutic resistance. Despite extensive efforts to characterize and target CSCs, their elusive nature continues to drive therapeutic resistance and relapse in epithelial malignancies. Single-cell RNA sequencing (scRNA-seq) has transformed our understanding of tumor biology. It enables high-resolution profiling of rare subpopulations (<5%) and reveals the functional heterogeneity that contributes to treatment failure. In this review, we discuss evolving evidence for a paradigm shift, enabled by rapidly advancing single-cell technologies, from a static, marker-based definition of CSCs to a dynamic and functional perspective. We explore how trajectory inference and spatial transcriptomics redefine stemness by context-dependent dynamic-state modelling. We also highlight emerging platforms, including artificial intelligence-driven predictive modelling, multi-omics integration, and functional CRISPR screens. These approaches have the potential to uncover new vulnerabilities in CSC populations. Together, these advances should lead to new precision medicine strategies for disrupting CSC plasticity, niche adaptation, and immune evasion.

## Graphical Abstract

**Graphical Abstract:**
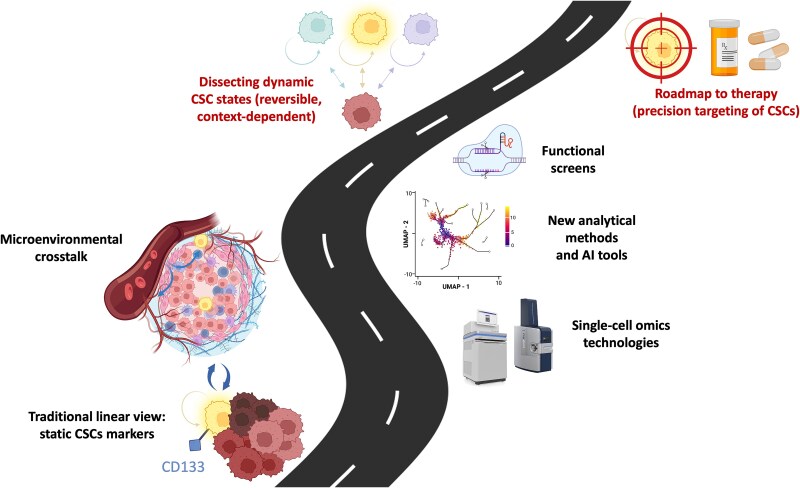

Illustration showing how modern single-cell profiling and bioinformatics tools, combined with functional screens, challenge the traditional static view of cancer stem cells and redefine them as a dynamic state. Alt text: Schematic of cancer stem cells redefined as a dynamic state.

## Introduction

Over five decades, the “cancer hallmarks framework” has codified core capabilities of cancer, yet it still does not fully account for systemic manifestations or the proximate causes of mortality [1]. Despite this logical scaffold, we lack a mechanistic account of how tumor-derived factors and host responses (immune, neuroendocrine, metabolic) converge to drive cachexia, coagulopathy, organ failure, and lethal relapse [2]. Future progress will hinge on mapping bidirectional communication between tumors and distant organs—spanning premetastatic niche formation, circulating and stromal intermediaries, and microbiota-host crosstalk—and situating these processes within environmental and physiological contexts [3–5]. This perspective advances a systems view of cancer, integrating tumor-intrinsic programs with host interactions across model systems and clinical datasets to guide prevention, risk stratification, treatment design, and ultimately improved patient outcomes [6].

“Cancer encompasses a diverse group of diseases” characterized by uncontrolled proliferation, genomic and epigenetic alterations, and the ability to invade and metastasize. Carcinomas, arising from epithelial tissues (in contrast to sarcomas, hematological malignancies, and neuroectodermal cancers), account for over 85% of adult cancers and represent the primary focus of this review [7]. Their incidence increases with age [8, 9], and in 2022, they constituted a substantial portion of the worldwide cancer burden [10]. Lung, breast, colorectal, prostate, and gastric carcinomas are among the most prevalent types, though incidence and mortality vary considerably by region and Human Development Index (HDI) [10]. Notably, the total number of new cancer cases is projected to increase sharply by 2050 [10], largely due to population growth, aging, and lifestyle-related risk factors, particularly in low- and middle-HDI countries, underscoring the urgent need for more effective prevention and control strategies [11].

Despite advances in diagnosis and treatment, patients with many epithelial malignancies continue to experience “dismal outcomes”. Pancreatic ductal adenocarcinoma (PDAC) remains the malignancy with an exceptionally poor prognosis, exhibiting an overall 5-year survival rate of <10% [12], primarily attributable to late-stage diagnosis [13] and its inherently aggressive tumor biology [12]. Even with comprehensive treatment strategies such as neoadjuvant therapy, 20.5% of patients achieve modest improvement in 5-year survival compared with 6.5% after upfront surgery [14, 15], underscoring the profound therapeutic challenges posed by this cancer type. For other diseases such as breast cancer, despite generally improving survival outcomes, the triple-negative subtype remains difficult to treat [16], which lacks targeted therapies and often exhibits treatment resistance [17, 18]. Likewise, lung cancer (e.g. small cell lung cancer) [19] and colorectal cancer [20] also continue to face persistent challenges due to high recurrence, metastasis, and acquired drug resistance.

**Figure 1:**
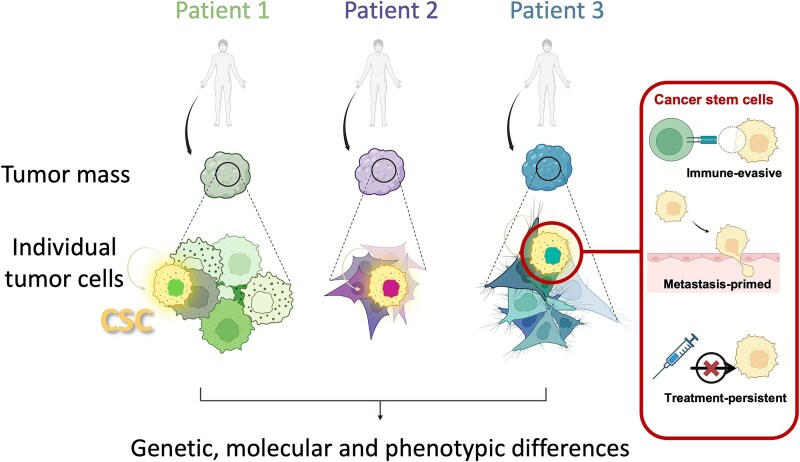
Tumor heterogeneity and the role of CSCs. Tumor heterogeneity occurs inter-tumorally (between patients). Distinct shades in the left panel illustrate inter-tumoral differences arising from genetic, epigenetic, and phenotypic variation. Tumor heterogeneity also occurs intra-tumorally (within individual tumours). Each tumor comprises diverse cellular subclones, some of which are dormant while others dominate the current clinical phenotype. Dormant clones are relatively quiescent populations that persist without driving growth, whereas dominant clones actively expand and shape the tumor’s behavior. Within each subclone, subsets of CSCs exhibit distinct though sometimes overlapping features, such as promoting tumor progression, driving metastatic dissemination, and resisting therapeutic interventions.

**Figure 2:**
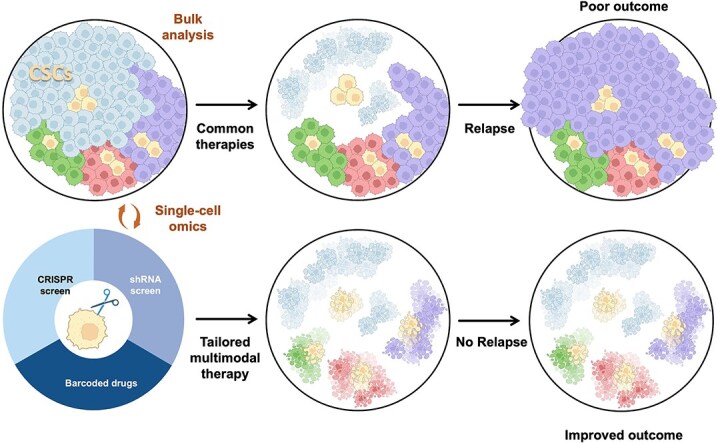
New approaches for studying tumor heterogeneity. Traditionally, tumors have been analyzed in bulk (“upper pathway”), which averages signals across mixed cell populations and therefore risks overlooking smaller subclones and, most critically, rare CSCs with immune-evasive, metastasis-primed, and treatment-persistent properties. This approach has mostly led to therapies that mainly target the dominant differentiated populations, allowing surviving CSCs to regenerate the tumor and contribute to relapse and poor outcome. By contrast, single-cell profiling (“lower pathway”) resolves rare clones and CSC subsets at high resolution. Integrated with CRISPR/shRNA screening and barcoded drug testing, these approaches uncover the molecular traits of rare, aggressive cells and guide the design of multimodal therapies—treatment strategies that combine conventional modalities with targeted interventions against resistant CSC populations. This improved understanding is contributing to the development of tailored multimodal therapies aimed at eradicating all malignant cells, including those with aggressive stem-like properties. Eventually, this comprehensive approach should result in fewer relapses and improved overall survival.

A primary driver of treatment failure and disease relapse is “tumor heterogeneity”, which reflects genetic, epigenetic, and microenvironmental diversity within and between tumors. This heterogeneity fosters the emergence of resistant subclones that survive therapy and contribute to recurrence (Fig. 1) [21]. Particularly challenging are rare—commonly representing <5% of the total cancer cell pool [22–24]–“cancer stem cells (CSCs)”, an aggressive subset of tumor cells defined by their capacity for self-renewal and plasticity that enables regeneration of heterogeneous lineages within the tumor. These cells often resist therapies through quiescence, drug efflux pumps, enhanced DNA damage repair, and immune evasion [25]. In addition to therapy resistance, CSCs fuel tumor progression by sustaining long-term growth through self-renewal, initiating metastasis via epithelial-mesenchymal transition (EMT)-driven plasticity and invasive programs, and fostering relapse after treatment through interactions with the tumor microenvironment (TME) [22, 25, 26]. Their role in tumor progression and therapy failure highlights the importance of clarifying the molecular and functional attributes of CSCs to inform more effective therapeutic strategies (Fig. 2). “Single-cell profiling” has now emerged as an indispensable tool for dissecting such cellular complexity. It allows the study of both rare clones and CSC subsets at high resolution. Combined with CRISPR/shRNA screening and barcoded drug testing, these approaches can reveal molecular traits of CSCs, enabling multimodal therapies that reduce relapse and improve survival.

**Figure 3:**
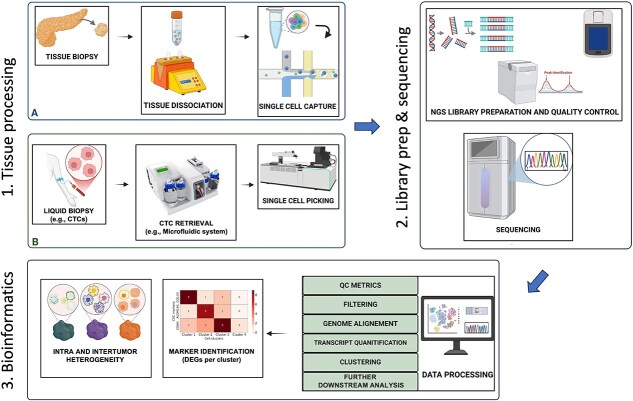
Single-cell transcriptomics workflow (clockwise). (i) Processing of tissue biopsies (A), followed by mechanical/enzymatic dissociation, or liquid biopsies (B), followed by CTC retrieval. Single-cell capture using droplets or microfluidic chips (e.g., 10x Genomics platform) or robotic cell picking systems (e.g., CellCelector, Sartorius) for isolating individual cells. (ii) Preparation of cDNA libraries by reverse transcription and next-generation sequencing. (iii) Data processing using bioinformatics pipelines (quality control, alignment, transcript quantification). For clustering and marker identification, data are converted into meaningful clusters or t-SNE/UMAP plots revealing rare cell types. Biomarker identification is further illustrated by a differential-expression heatmap showing genes enriched in distinct clusters, and heterogeneity analysis highlights both intra- and inter-tumoral diversity.

Standardized single-cell processing workflows have been developed to dissect both tissue and liquid biopsies using droplet/microfluidic platforms or robotic picking (Fig. 3). Reproducible bioinformatics pipelines (QC, alignment, quantification) generate high-quality expression matrices that enable downstream analyses such as clustering and dimensionality reduction (Uniform Manifold Approximation and Projection [UMAP]/t-SNE) to delineate cellular states and uncover rare populations. As such, single-cell profiling has become critical for profiling cell states in both healthy and diseased tissues [27]. Specifically, single-cell RNA-seq (scRNA-seq) allows the amplification of the complete transcriptome from individual cells, providing high-resolution insights into gene expression states [27] and enabling the study of inter- and intra-tumoral heterogeneity. Biomarkers for distinct subsets of cells can be determined by differentially expressed genes across transcriptionally defined clusters. These genes nominate candidate CSC markers that can subsequently be validated in functional assays (Fig. 3).

Notably, through newly emerging scRNA-seq studies the underlying concept that CSCs as rare but static entities has been challenged, suggesting that “stemness might be a rather dynamic, context-dependent state” [28]. Consistent with this, Rhim *et al.* demonstrated in PDAC mouse models that cancer cells undergoing EMT acquire stem-like properties, including enhanced tumor-initiating potential, illustrating that stemness can be acquired, potentially transient, and influenced by microenvironmental context [29].

With the expanding scale of single-cell transcriptomic data, a series of computational frameworks based on high-dimensional gene expression patterns have emerged. “New bioinformatics tools” enable us to infer cellular differentiation potential, state transition rates, and even fate decisions—without relying on traditional surface markers. Among them, methods such as transcriptional entropy (quantifying the degree of “disorder” or “uncertainty” in a cell by computing the entropy of its transcriptome, as an indicator of its differentiation potential or phenotypic plasticity) and RNA velocity (predicting immediate future states from unspliced/spliced mRNA ratios, assuming steady-state kinetics) are free of external cell-type labels; while some stemness scoring tools (e.g. mRNAsi, StemSC), though also independent of traditional surface markers, rely on training with stem cell reference samples and thus fall under supervised categories (detailed in Section 4) (Table 1). Collectively, these approaches now enable the dynamic characterization of CSC potential at unprecedented resolution.

**Table 1:** Bioinformatic tools for assessing stemness.

Tool	Algorithm	Platform	URL	Reference
StemID	Shannon entropy	R	https://github.com/dgrun/StemID	[85]
SCENT SR	Signaling entropy	R	https://github.com/aet21/SCENT	[75]
SLICE	Single-cell entropy	R	http://research.cchmc.org/pbge/slice.html	[71]
mRNAsi	Machine learning	R, web server	https://bioinformaticsfmrp.github.io/PanCanStem_Web/	[80]
scEpath	Inference of transition probabilities	MATLAB	https://github.com/sqjin/scEpath	[81]
CytoTRACE	Gene counts and expression	R, web server	https://cytotrace.stanford.edu/	[77]
SCENT CCAT	Correlation of connectome and transcriptome	R	https://github.com/aet21/SCENT	[74]
scCancer	Machine learning	R	https://github.com/wguo-research/scCancer	[82]
StemSC	Relative expression orderings of gene pairs	R	https://github.com/Zhao-Wenyuan/StemSC	[83]
FitDevo	Sample-specific gene weight	R	https://github.com/jumphone/fitdevo	[84]
Cancer StemID	TF regulatory activity estimation	R	https://github.com/aet21/CancerStemID	[85]
SPIDE	Cell-specific network entropy	Python	https://github.com/CSUBioGroup/SPIDE	[73]
CytoTRACE2	Deep learning	R, Python	https://github.com/digitalcytometry/cytotrace2	[77]
Cancer Stemness Online	Integration of existing algorithms	Web server	http://bio-bigdata.hrbmu.edu.cn/CancerStemnessOnline/	[86]

Supported by these advances, we propose that CSCs represent reversible states along developmental and treatment-induced trajectories rather than a fixed, intrinsic phenotype. We further argue that the most promising therapeutic strategies may not target static CSC markers but instead exploit transient, high-entropy states during cell state transitions—periods of instability that may represent therapeutic opportunities. Furthermore, we advocate for functional single-cell perturbation assays as the gold standard for identifying CSC vulnerabilities, and propose a typology of CSCs based on their role in the tumor ecosystem—such as immune-evasive, metastasis-primed, or therapy-persistent states. Ultimately, we envision a cross-cancer CSC plasticity atlas that integrates single-cell profiling with spatial and functional omics to guide future therapeutic strategies. Our review builds on a growing body of literature and integrates it with novel insights to provide a forward-looking roadmap for both basic and translational CSC research.

## Beyond established cancer stem cell biomarkers: a quest for a new definition

Historically, CSCs have been defined by a set of “phenotypic biomarkers” [30], including CD133 [22, 31, 32], CD44 [26, 33, 34], and ALDH1 [35, 36]. CSCs co-express multiple surface markers, reflecting their phenotypic heterogeneity, as exemplified in colon cancer [37], hepatocellular carcinoma [38], and pancreatic cancer [22]. However, mounting evidence suggests these markers are neither exclusive to CSCs nor consistent across tumor types or conditions [39]. For instance, CD133 was initially identified as a hematopoietic stem cell marker [40], but is now widely used as a CSC marker in various cancers, including breast, prostate, colon, liver, lung, and pancreas tumors [41, 42]; CD44, one of the first CSC biomarkers, is involved in cell adhesion and migration in both normal and malignant tissues [43].

By resolving transcriptional states at single-cell resolution, these technologies challenge the static marker-based definition of CSCs. Newly emerging data using single-cell transcriptomic technologies have revealed that CSC identity might be better represented as a fluid spectrum of epigenetically regulated transcriptional states, further modulated by niche interactions, environmental stress, and therapy-induced plasticity [44, 45]. This paradigm shift is powerfully illustrated by a single-cell multi-omics study in bladder cancer [46]. Although recurrent tumors were enriched in cells expressing conventional CSC markers like CD44 and ALDH1A1, scRNA-seq revealed that these markers were not exclusive to a discrete cluster but were heterogeneously expressed across a spectrum of cell states [46]. Critically, the functional stemness capacity was governed by dynamic epigenetic regulators such as EZH2 and KDM5B [46].

By integrating scRNA-seq with single-cell assay for transposase-accessible chromatin using sequencing (scATAC-seq) profiles from primary tumors—followed by bulk CUT&Tag and ATAC-seq after EZH2 knockdown—the study showed that the CSC subpopulation enriched in recurrent cancers sustains stemness through EZH2-mediated deposition of H3K27me3 that keeps the tumor-suppressive cell-adhesion gene NCAM1 transcriptionally silent [46]. The cell-type-specific correlation between reduced chromatin accessibility at the NCAM1 promoter and low NCAM1 expression in the same CSC subset provides compelling evidence that CSC identity is governed by dynamic epigenetic reprogramming [46]. Functionally, loss of EZH2 led to genome-wide loss of H3K27me3, gain of H3K27ac, and de-repression of NCAM1, thereby impairing self-renewal and tumorigenicity [46].

Similarly, “the dynamic nature of CSCs” is further regulated by the TME. Using integrated single-cell and spatial transcriptomics, Zhang *et al.* [45] captured the transition of tumor cells from a spasmolytic polypeptide–expressing metaplasia (SPEM) state to a CSC state, largely driven by inflammatory cancer-associated fibroblasts (iCAFs) secreting AREG in the TME. Ligand-receptor analysis and functional validation (via AREG/ERBB2 knockdown and lapatinib treatment) demonstrated that this niche cue activates ERBB2–AKT signaling, leading to upregulation of SOX9 and OLFM4, which enhances stemness and chemoresistance, as evidenced by increased sphere formation and drug resistance *in vitro*. This study provides direct evidence that CSC identity is a transient, TME-induced transcriptional state rather than a fixed cellular entity.

Therefore, CSCs should be redefined as dynamic entities whose identity is shaped by intrinsic plasticity and microenvironmental cues, rather than fixed surface markers. These findings challenge the utility of static surface markers for defining CSCs and underscore the necessity of understanding the dynamic epigenetic mechanisms that underlie functional stemness.

## The tumor ecosystem and cancer stem cell states: lessons from single-cell transcriptomics studies

**Figure 4:**
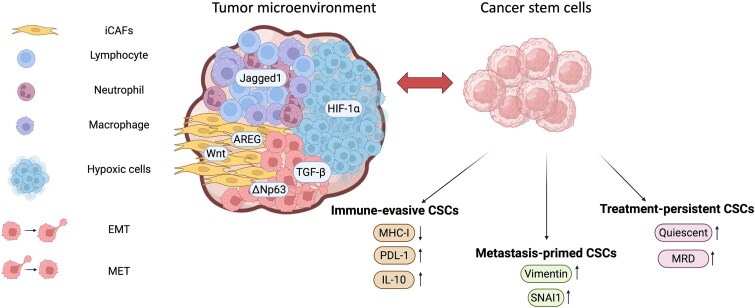
CSC plasticity in the tumor microenvironment. CSCs exist in dynamic states shaped by signals from the tumor niche, including inflammatory CAFs, immune cells, and hypoxia. These cues drive reversible transitions into immune-evasive, metastasis-primed, or treatment-persistent CSCs. Key pathways include WNT, AREG, Jagged1–Notch, TGF-β, and HIF-1α (hypoxia-inducible factor-1α), highlighting the role of the microenvironment in promoting therapy resistance and metastatic potential. Immune-evasive CSCs downregulate MHC-I, upregulate PD-L1, and secrete IL-10. Metastasis-primed CSCs show increased Vimentin and SNAI1, while treatment-persistent CSCs are linked to quiescence and enrichment in minimal residual disease (MRD). Both EMT and MET states are indicated, with the two cell icons denoting the EMT process and illustrating how cells transition through this state.

“Spatial and trajectory analyses of single-cell profiles” are computational approaches that map the physical organization of cells within tissues and reconstruct their dynamic developmental or treatment-induced transitions over time. These methods have uncovered how CSC states fluctuate in response to microenvironmental cues and therapeutic pressures (Fig. 4) [45]. CSCs do not exist in isolation but are embedded in a complex tumor ecosystem that includes CAFs, various types of immune cells, and hypoxic or nutrient-deprived niches. Spatial cues regulate stemness through paracrine signaling, e.g. AREG-ERBB2 and WNT signaling derived from iCAFs with low α-SMA expression, driving tumor progression and therapy resistance [45].

Disruption of these spatial or microenvironmental cues can attenuate stemness; e.g. interfering with ROS-related pathways sensitizes CSCs to radiotherapy [47], while blockade of Jagged1 reduces dormant CSC populations and delays recurrence [48]. Importantly, niche-induced stemness is reversible—suggesting that CSCs may arise not solely from rare clones but also dynamically from non-CSC states under the appropriate selective pressure [49]. For instance, in EMT states, even the most mesenchymal tumor cells can revert to epithelial phenotypes (MET) in the lung microenvironment, though metastasis is driven primarily by hybrid EMT states [50]. This plasticity is regulated by factors such as ΔNp63 (epithelial maintenance) and TGF-β (mesenchymal promotion) [50], further supporting the concept that stemness is a transient, context-dependent state. Building on this framework, we propose a functional typology of CSCs.

First, “immune-evasive CSCs” actively suppress immune recognition by downregulating MHC-I, upregulating immune checkpoint molecules such as PD-L1, or secreting immunosuppressive cytokines such as IL-10 [51]. This is supported by the identification of a CSC signature through single-cell pseudotime analysis (Slingshot [52]), which positioned CSCs at the origin of a differentiation trajectory and revealed suppressed immune-related pathways [53]. Spatial transcriptomics further validated their immune-evasive phenotype by demonstrating their co-localization with osteopontin-expressing SPP1^+^ (secreted phosphoprotein 1, also known as osteopontin) macrophages in hypoxic tumor regions that are devoid of CD8^+^ T cells and enriched for the immune checkpoint molecule HAVCR2 (TIM-3).

Second, “metastasis-primed CSCs” express signatures of EMT, thereby exhibiting enhanced motility and invasiveness. Indeed, Nishiyama *et al.* demonstrated that hepatoma CSCs with upregulated EMT markers (Vimentin and SNAI1) and activation of EMT-related pathways promoted liver metastasis [54], consistent with the broader role of EMT in driving metastatic dissemination. Building upon this, Zhou *et al.* not only identified a CSC subpopulation with high EMT activity through transcriptional clustering, but also employed single-cell pseudotime trajectory analysis (by Monocle2 [55]) to reconstruct a “stem-to-invasion” path [56]. This trajectory revealed a continuous transcriptional transition from CSCs with stem-like properties to cells exhibiting enhanced invasive potential, providing dynamic evidence that these CSCs are primed for metastasis.

Third, “treatment-persistent CSCs” are those that survive chemotherapy, radiation, or targeted therapy through various mechanisms including quiescence, efflux pump expression, enhanced DNA damage repair, and immune evasion [47, 57]. While some of these cells represent inherently resistant subclones, others may arise via dedifferentiation of non-CSCs under drug-induced stress [57]. Their persistence might be transient but clinically challenging as they can serve as a reservoir for disease relapse. Indeed, Nojima *et al.* [58] identified a subpopulation of treatment-resistant cancer stem-like cells in colorectal cancer organoids. Through scRNA-seq, RNA velocity (by scVelo [59]) and trajectory inference (TI) analyses (by Monocle3 [60]), the study identified that these cells, particularly the TC1 cluster in malignant organoids, originate from stem cells and exhibit elevated expression of Wnt signaling and CSC marker genes (e.g. SOX4, PERP, TESC). This pseudotemporal reconstruction supported their stem-like origin and persistence phenotype. Furthermore, machine learning (ML) models trained on public persister cell data classified these TC1 cells as drug-tolerant persisters (DTPs), and functional assays confirmed synergistic effects of YM-155 (survivin inhibitor) and THZ2 (CDK7 inhibitor) with trametinib (MEK inhibitor), providing a combinatorial strategy to target these resistant cells.

scRNA-seq has also been instrumental in characterizing dormant CSCs, which persist after therapy as minimal residual disease (MRD). In a breast cancer model of MRD, Janghorban *et al.* performed scRNA-seq across four tumor states: primary, dormant, long-term dormant, and recurrent tumors [48]. TI (by Monocle [61]) revealed a dynamic transition of cancer cells from a proliferative state through dormancy and toward recurrence, uncovering a distinct, dormant CSC cluster (CC6). Among transcriptionally distinct CSC clusters, Notch3 and Notch4 signaling was specifically enriched in the dormant populations. The immune microenvironment in dormant and long-term dormant tumors remained immunosuppressive, characterized by myeloid-derived suppressor cells expressing the Notch ligand Jagged1. Functional blockade of Jagged1 using a neutralizing antibody during dormancy significantly reduced the dormant CSC population and delayed recurrence. Consistent with this, pharmacological inhibition of Notch1 has also been shown to suppress CSC features and limit tumor growth in preclinical models [62]. Together, these findings highlight Notch signaling as a potential therapeutic vulnerability in CSCs.

## Rewriting cancer stem cell biology: cancer cell stemness and pseudotime as functional markers

**Figure 5:**
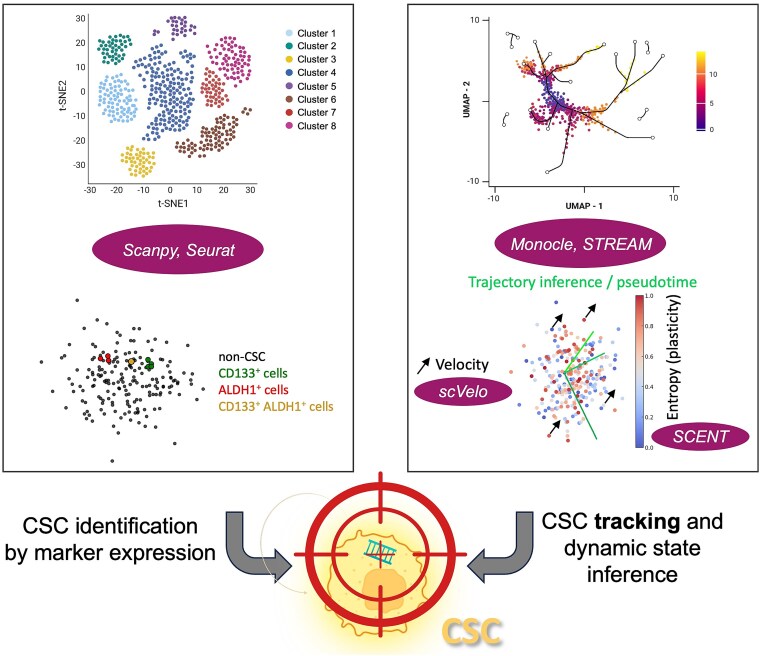
Static clustering and dynamic trajectories in single-cell dissection of CSCs. Static identification methods, where t-SNE-based clustering (SCDE, DGEclust, Seurat) is combined with gene markers that enable discrete CSC subpopulation mapping and biomarker discovery (left panel). Dynamic tracking capabilities, employing UMAP visualization with Monocle/STREAM for pseudotemporal ordering, scVelo for RNA velocity analysis, and SCENT for entropy-based stemness quantification, are able to reveal CSC state transitions, trajectory dynamics, and stemness evolution (right panel).

With the rapid advancement of biotechnology, single-cell profiling now allows us to move beyond static snapshots and study how cells change over time (Fig. 5). This process, known as TI, aims to computationally reconstruct the paths that cells likely follow during dynamic transitions, such as acquisition of stemness (dedifferentiation), unilateral or even multilateral differentiation, or therapy resistance [63]. Unlike bulk sequencing, which provides a population-level average, single-cell data enables inference of lineage relationships by comparing transcriptional similarity among thousands of individual cells [64]. These inferred trajectories can reveal how CSC states emerge, stabilize, or evolve under selective pressures and/or tumor progression. One core approach is pseudotime analysis [61], which arranges cells along a virtual trajectory, suggesting how stem-like cells may differentiate or revert. Conventional approaches that rely on a priori cell labelling or bulk analysis are susceptible to synchronization bias, where the averaging of unsynchronized cells obscures true temporal dynamics and can lead to misleading conclusions—a phenomenon known as Simpson’s paradox [61]. This can severely impact downstream analyses by distorting inferred gene–gene relationships and masking the continuous waves of transcriptional regulation that occur at the single-cell level [61].

Tools like “Monocle” [61] and “STREAM” [65] infer these paths prospectively, without prior cell labelling, thereby preserving the intrinsic heterogeneity of the process. More specifically, Monocle reconstructs single-cell trajectories by constructing a minimum spanning tree (MST) on dimension-reduced expression data and then ordering cells along the longest path of the MST, while STREAM [65] employs Elastic Principal Graphs to infer branching trajectories by optimizing graph topology under structural constraints in reduced-dimensional space. Subsequently, the developed Monocle2 [55] also enables the unsupervised inference of such trajectories. By introducing Reversed Graph Embedding and the DDRTree dimensionality reduction method, it improves computational efficiency and scalability when dealing with large numbers of cells, significantly enhancing the ability to identify branching and multifate trajectories [55].

Compared with Monocle2, “Monocle3” [60] replaces t-SNE with UMAP, which better preserves global geometry. While t-SNE (t-distributed Stochastic Neighbor Embedding) has been instrumental in visualizing local cell–cell relationships, it often distorts broader topology and fragments trajectories into seemingly disconnected clusters. UMAP overcomes these limitations by maintaining both local neighborhoods and global geometry, providing a more faithful representation of developmental and differentiation landscapes. Monocle 3 also replaces the single MST trajectory with PAGA-guided principal-graph learning [66], enabling support for disconnected components, loops, and branching or converging lineages. Further advances include landmark-based graph construction to increase the cell-number ceiling, as well as automatic branch pruning, loop closure, and projection-based pseudotime, together yielding more accurate and biologically realistic trajectories.

In a study of early gastric cardia adenocarcinoma, Wang *et al.* successfully reconstructed the trajectory of transformation from normal pit mucous cells (PMCs) and gland mucous cells to malignant epithelial cells using Monocle2 [67]. This not only revealed the activation dynamics of pathways such as WNT and NF-κB during malignant transformation but also further identified NNMT as a key driver gene. Its high expression in aquaporin 5^+^ (AQP5) CSCs maintains cell stemness by regulating histone methylation, thereby promoting malignant tumor progression [67]. This study fully demonstrates the powerful capability of unsupervised TI methods in uncovering the dynamic evolution mechanisms of CSCs.

A related concept is “RNA velocity”, which uses the ratio of newly transcribed (unspliced) to mature (spliced) RNA to predict the cells’ potential future states [68]. Unspliced and spliced RNA molecules are distinguished based on the presence or absence of intronic sequences, which can be captured by common scRNA-seq protocols that use oligo-dT primers (such as SMART-seq2, STRT/C1, inDrop, and 10x Genomics Chromium), which unexpectedly capture a substantial fraction (15%–25%) of intronic reads due to mechanisms like internal priming within introns [68]. If the expression of specific genes is rising, RNA velocity can illustrate the “arrow” pointing toward the corresponding cell fate. This reveals not only where a CSC is right now, but also where it might be going.

For instance, in a scRNA-seq study of collecting duct renal cell carcinoma (CDRCC), RNA velocity and pseudotemporal trajectory analyses revealed that a novel CSC subpopulation functions as a differentiation hub, sequentially giving rise to primary tumor cells (Cancer 1/3), lymph node metastatic cells (Cancer 2), and bone metastatic cells (Cancer 4) in a spatially and temporally ordered manner [69]. This approach not only confirmed the stemness properties—such as self-renewal and multilineage differentiation—of CSCs, but also elucidated their pivotal role in promoting osteolytic bone metastasis through a positive feedback loop mediated by FGF2/FGFR1 and FGF7/FGFR3 ligand-receptor interactions between CSCs and osteoclasts within the bone microenvironment [69].

Table 1 summarizes 14 common and dedicated software or web servers for assessing cell stemness. Most of them are based on transcriptional entropy and ML algorithms. A fundamental principle underlying entropy-based approaches is that cells with high stemness exhibit a relatively uniform transcriptome, in which many genes are expressed at comparable levels. By contrast, more differentiated cells display a restricted transcriptome, characterized by a limited set of genes expressed at markedly elevated levels. Building on this concept, several entropy-based methods have been developed to quantify cellular differentiation potential, including StemID, SLICE, SCENT, and SPIDE [70–73]. These approaches have demonstrated robustness and broad applicability, as evidenced by their successful application and validation across multiple independent datasets with well-defined lineage hierarchies and developmental time information.

“StemID” builds upon this concept by assessing cellular stemness through the calculation of a cell’s transcriptome Shannon entropy [70].

“SPIDE” demonstrates strong accuracy and reliability by constructing cell-specific protein–protein interaction (PPI) networks and reducing bias caused by dropout events through expression smoothing techniques [73].

“SLICE” estimates the differentiation state of cells by calculating single-cell entropy based on the diversity of gene expression patterns and functional activation probabilities [71].

Another powerful metric is signaling entropy introduced by “SCENT”, a sophisticated form of transcriptional entropy that measures a cell’s “undecidedness” or “plasticity” within the constraints of a PPI network [72, 74, 75]. SCENT computes the entropy of signal flow during a random walk on the PPI network, where genes that are highly expressed and centrally positioned in signaling pathways exert a greater influence. Cells with high signaling entropy exhibit broad fate potential and are typically found in stem-like or transitional states, whereas low signaling entropy reflects commitment, with signaling restricted to a few pathways and lineage-specific genes expressed in a differentiated program [72, 75]. This state suggests a more differentiated, specialized fate. For instance, in circulating tumor cells (CTCs) from patients with metastatic prostate cancer, high signaling entropy significantly enriched for a subpopulation resistant to enzalutamide and marked by ALDH7A1 expression—a known CSC marker. This underscores the utility of SCENT in pinpointing malignant subpopulations with enhanced plasticity and therapy resistance, directly from single-cell transcriptomes without prior feature selection. And for a fast and accurate stemness estimation, a new single-cell potency measure, correlation of connectome and transcriptome (CCAT) was developed within SCENT [72, 74].

Collectively, SPIDE constructs cell-specific PPI networks to reduce bias, while SLICE does not directly utilize PPI networks but instead focuses on gene expression patterns and functional activation probabilities [71, 73, 76]. Unlike SCENT, which uses the entire transcriptome without feature selection, SPIDE and SLICE may rely on specific gene features or functional modules to varying degrees [71–73, 76]. To handle dropout events, SPIDE employs expression smoothing techniques, SCENT indirectly mitigates the impact of dropouts through signaling entropy calculation, and SLICE addresses this issue by emphasizing gene expression diversity [71–73, 76]. Collectively, these methods differ in their network utilization, feature selection strategies, and approaches to handling dropout events, reflecting diverse philosophies in estimating cell differentiation states.

The principle of “CytoTRACE” primarily relies on the correlation between gene expression levels and the number of expressed genes in a cell to estimate its stemness. CytoTRACE can evaluate the differentiation state of cells in single-cell data without requiring prior knowledge, leveraging the Gene Counts Signature [77]. It assigns a score to each cell, with higher scores indicating greater stemness. This robust algorithm, validated on large datasets, outperforms previous stemness prediction methods [77]. The CytoTRACE calculates scores ranging from 0 to 1, where higher scores correspond to less differentiation. Zhang *et al.* utilized the CytoTRACE algorithm to evaluate the stemness of cancer cells and, based on this, revealed a pan-cancer stemness signature (Stem.Sig) that predicts responses to immunotherapy [78]. CytoTRACE is widely utilized; and then CytoTRACE2 [79] was developed based on an interpretable deep learning (DL) framework, enabling the prediction of classically defined cell potency labels and differentiation states on an absolute scale from scRNA-seq data.

In addition, “mRNAsi” utilizes a one-class logistic regression (OCLR) ML algorithm, training varying degrees of stemness signatures from publicly available molecular profiles [80]. “scEpath” computes energy landscapes and probabilistic directed graphs to reconstruct developmental trajectories, providing an algorithm for integrating “single-cell energy” and distance-based metrics [81]. “scCancer” is a specialized software designed for the processing and analysis of scRNA-seq data in cancer research [82]. It incorporates several cancer-specific features, including comprehensive quality control metrics, identification of major cancer microenvironment cell populations, and estimation of malignancy and stemness scores. The stemness score function in scCancer is like mRNAsi, utilizing an OCLR algorithm.

“StemSC” is grounded in the relative expression orderings (REOs) of gene pairs. In brief, StemSC first identifies stemness-related genes by selecting those significantly correlated with differentiation time [83]. Next, it establishes reference REOs using 13 RNA-seq datasets derived from both bulk and single-cell embryonic stem cell (ESC) samples. Ultimately, the StemSC value for a given sample is computed as the proportion of gene pairs that maintain the same REOs as those observed in the ESC samples. “FitDevo” is a method designed to infer developmental potential from scRNA-seq data. Its core approach involves generating sample-specific gene weights (SSGW) and then calculating the correlation between SSGW and gene expression to estimate developmental potential [84].

“CancerStemID” is a computational method that estimates the stemness index of cells from single-cell RNA sequencing data by measuring the regulatory activity of transcription factors (TFs) that control differentiation within a cell lineage [85]. The core hypothesis is that the number of tissue-specific TFs displaying low differentiation activity in a given cell is a marker of stemness and cancer risk. In the context of esophageal squamous cell carcinoma, CancerStemID was extensively validated and demonstrated its ability to identify undifferentiated preneoplastic cells whose transcriptomic state is overrepresented in invasive cancer [85]. By analyzing the differentiation activity of tissue-specific TFs, CancerStemID can pinpoint cells that exhibit high stemness and are at a higher risk of progressing to cancer, thus providing a novel computational strategy for early detection and risk prediction of cancer. Finally, “Cancer Stemness Online” [86] is a web-based platform that serves as a comprehensive resource for evaluating cancer stemness potential across both bulk and single-cell levels. This platform consolidates eight robust predictive algorithms including unsupervised and supervised methods, along with 27 signature gene sets linked to cancer stemness, enabling accurate prediction of stemness scores.

Together, studies using bioinformatic tools such as TI, RNA velocity, and entropy shift the definition of stemness from a fixed identity to a transitional, context-dependent state, shaped by epigenetic rewiring in response to the TME, spontaneous tumor progression, and selective pressure during therapy.

## Emerging single-cell omics studies on dissecting cancer stem cell landscapes

We next highlight representative studies across different cancer types that have applied single-cell omics to dissect CSC heterogeneity. A comprehensive list of studies with summaries and accession numbers is provided in Table 2, while the following paragraphs discuss selected examples in more detail to illustrate how these approaches uncover regulatory programs and therapeutic vulnerabilities shaping our understanding of CSC biology.

**Table 2:** Single-cell datasets across carcinomas for CSC atlas construction.

Type	Dataset	Accession number	Reference
Colorectal cancer	snRNA-seq and scATAC-seq of human colorectal polyps, normal colon tissues, and colorectal cancers	GSE201349	[87]
Liver cancer	scRNA-seq of hepatocellular carcinoma (HCC) primary tumors, portal vein tumor thrombus (PVTT), metastatic lymph node and non-tumor liver tissues	GSE149614	[88]
Bladder cancer	scRNA-seq and scATAC-seq of human low recurrence risk, high recurrence risk, and recurrent bladder cancer	HRA001088	[46]
Pancreatic cancer	scRNA-seq of primary PDAC tumors and normal pancreas	PRJCA001063	[90]
Gastric cancer	scRNA-seq of primary gastric tumors, adjacent non-tumor tissues, and organ-specific metastases (liver, peritoneum, ovary, lymph node)	n.a.	[91]
Breast cancer	scRNA-seq of primary breast tumors, dormant tumors, long-term dormant tumours, recurrent tumors, and treatment arms	GSE171464	[48]
Renal cell carcinoma	scRNA-seq of primary tumors and metastatic tumors of CDRCC	n.a.	[69]

Note: Accession numbers correspond to the original source of the single-cell data provided in indicated references.

n.a. = not available as the authors did not provide an accession number or a standard Data Availability Statement in the manuscript. Details on data generation are described in the respective Methods section.

**Figure 6:**
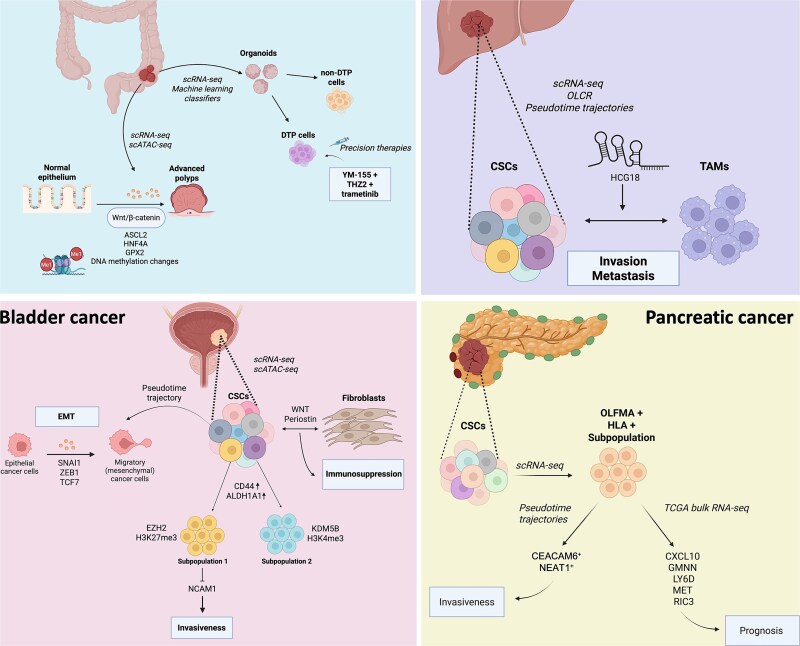
Dissecting cancer heterogeneity for various carcinomas. scRNA-seq and complementary single-cell omics approaches were used to unravel CSC heterogeneity, regulatory networks, and microenvironmental interactions across different solid tumours. “In colorectal cancer” (top left), scRNA-seq and scATAC-seq explored the transition from normal epithelium to advanced polyps and carcinoma. This revealed WNT/β-catenin activation, ASCL2, HNF4A, and GPX2 as key stemness regulators, together with DNA-methylation changes that mirrored chromatin accessibility alterations and suggested potential early detection biomarkers. DTP cells identified in organoid models could be targeted by precision therapies such as YM-155, THZ2, and trametinib. “In liver cancer” (top right), scRNA-seq with OCLR scoring identified CSCs interacting with tumor-associated macrophages, where lncRNA HCG18 promotes invasion and metastasis via CSC–immune crosstalk. “In bladder cancer” (bottom left), scRNA-seq and scATAC-seq defined two epigenetically distinct CSC subpopulations regulated by EZH2 (H3K27me3) and KDM5B (H3K4me3), with EZH2 repressing NCAM1 to promote invasiveness. EMT trajectories are driven by TCF7, SNAI1, and ZEB1, while WNT and Periostin signaling mediate fibroblast-induced immunosuppression. “In pancreatic cancer” (bottom right), scRNA-seq and pseudotime analysis reveal an invasive CSC subpopulation marked by OLFM4 and HLA. Transition into CEACAM6^+^ and NEAT1^+^ states correlates with invasiveness. Integration with TCGA data identifies five CSC-associated genes (e.g. MET, LY6D) linked to poor prognosis. Together, these panels highlight how single-cell omics dissects CSC heterogeneity, dynamics, and vulnerabilities across tumor types.

In “colorectal cancer”, single-nucleus RNA sequencing (snRNA-seq) and scATAC-seq, a method that profiles chromatin accessibility at single-cell resolution, were employed to chart the molecular and cellular continuum from normal colon epithelium to precancerous polyps and invasive carcinoma (Fig. 6) [87]. Stem-like epithelial cells displayed progressive activation of WNT/β-catenin signaling via TCF/LEF motifs and ASCL2, coupled with loss of KLF and HOX-family motifs, with these transcriptional programs closely mirrored by changes in chromatin accessibility revealed by scATAC-seq, underscoring coordinated transcriptional and epigenetic regulation. Advanced polyps were characterized by expanded stem-like populations, regulatory T cells, and preCAFs, whereas established tumors exhibited exhausted T cells and RUNX1-regulated CAFs. Key findings included GPX2 as an early oxidative-stress mediator and HNF4A as a driver of malignant transformation. Moreover, DNA methylation was inversely correlated with chromatin accessibility changes: regions gaining accessibility tended to exhibit hypomethylation, while closed chromatin regions were often hypermethylated. However, this relationship was not absolute, a subset of loci showed methylation-independent accessibility changes, suggesting additional regulatory layers such as histone modifications or TF occupancy. These observations pinpoint ASCL2, HNF4A, and GPX2 as critical regulatory nodes whose modulation could disrupt CSC maintenance and immune evasion, potentially intercepting colorectal cancer progression at the polyp stage.

A complementary investigation integrated scRNA-seq with ML classifiers to identify treatment-resistant cells in organoids derived from familial adenomatous polyposis patients [58]. These classifiers were trained on scRNA-seq gene expression profiles, extracting transcriptional features to distinguish DTP cells—a defining feature of CSCs—from non-DTPs, and their predictions were validated by *in vitro* drug-response assays. The models distinguished DTPs from non-DTPs and enabled *in silico* drug screening. A TC1 cluster enriched for DTP traits emerged, and combinatorial regimens incorporating YM-155 (survivin inhibitor), THZ2 (CDK7 inhibitor), and trametinib (MEK inhibitor) were prioritized. *In vitro*, YM-155 or THZ2 synergized with trametinib, revealing DTP-specific vulnerabilities and providing a rationale for precision combination therapies in CRC.

In “liver cancer”, scRNA-seq integrated with stemness scoring based on the OCLR ML algorithm and pseudotime trajectories elucidated how lncRNA HCG18 orchestrates vascular invasion by modulating CSC-macrophage interactions [88]. The OCLR algorithm establishes a prediction model using pluripotent stem cell samples (ESC and iPSC) from the PCBC dataset to quantify stem-like transcriptional features. This score does not rely on the expression levels of specific marker genes but is instead a computational metric derived from transcriptomic data that reflects the degree of tumor undifferentiation or stem-like characteristics. This integrative approach offers a systematic framework for targeting CSC-driven invasion and metastasis (Fig. 6).

In “bladder cancer”, scRNA-seq and scATAC-seq have delineated the dynamic epigenetic and transcriptional architecture of CSCs during tumor recurrence [46]. Single-cell analysis in this work uncovered a heterogeneous CSC pool enriched in recurrent tumors, distinguished by elevated CD44 and ALDH1A1 expression together with the epigenetic regulators EZH2, the catalytic subunit of the polycomb repressive complex 2, and KDM5B, a histone H3K4 demethylase. Although H3K4me3 and H3K27me3 were not profiled at the single-cell level, subsequent bulk CUT&Tag for these modifications in EZH2-knockdown cell lines, integrated with the scRNA-seq and scATAC-seq data, suggested that the two regulators mark distinct CSC subpopulations: one controlled by KDM5B-mediated H3K4me3 demethylation and another driven by EZH2-dependent H3K27me3 repression [46]. Crucially, EZH2 maintained stemness by silencing the cell-adhesion molecule NCAM1, thereby fostering invasiveness. EZH2-mediated silencing of NCAM1 was attributed to increased deposition of H3K27me3 at the NCAM1 promoter, as shown by bulk ATAC-seq footprinting and bulk CUT&Tag analyses. This repressive chromatin state was further associated with local CpG hypermethylation, suggesting a cooperative epigenetic mechanism maintaining NCAM1 suppression and CSC invasiveness [46, 89]. EZH2 knockdown by shRNA induced NCAM1 expression, lowered CD44 and N-cadherin, and attenuated xenograft growth (Fig. 6) [46].

Pseudotemporal trajectory reconstruction exposed a continuous EMT program, with TCF7 emerging as a pivotal regulator alongside classic EMT inducers SNAI1 and ZEB1. scATAC-seq footprinting confirmed increased TCF7 motif accessibility in EMT-progressing cells, while functional assays showed that TCF7 knockdown curtailed migration, invasion, and tumor expansion by reducing mesenchymal markers VIM and SNAIL1. Cell–cell communication analysis further identified noncanonical WNT and Periostin signaling as recurrence-specific pathways mediating CSC-CAF crosstalk, thereby recapitulating the immunosuppressive niche of advanced disease. Together, these data highlight the interplay between epigenetic control (EZH2/KDM5B), transcriptional reprogramming (TCF7-driven EMT), and stromal signals in shaping bladder-CSC plasticity, nominating EZH2 and TCF7 as therapeutic entry points to prevent recurrence and metastasis.

In “pancreatic cancer”, scRNA-seq interrogated ductal-cell heterogeneity and invasive trajectories, identifying a CSC-like subpopulation (Cluster 2) marked by heightened OLFM4 and HLA expression [90]. Pseudotime analysis traced its transition into invasive clusters 3–5 expressing CEACAM6 and NEAT1. Integrating TCGA bulk-RNA profiles yielded five CSC-related prognostic genes (CXCL10, GMNN, LY6D, MET, RIC3). Elevated mesenchymal–epithelial transition (MET) and LY6D protein levels in tumor tissue underscore their translational potential (Fig. 6). Collectively, these insights spotlight CSC-like ductal cells as key PDAC drivers and nominate MET and LY6D for CSC-targeted intervention, with the CSC signature correlating strongly with tumor-mutation burden.

Finally, in “gastric cancer”, single-cell profiling combined with ML analyses unraveled the transcriptional heterogeneity of organ-specific metastasis [91]. The authors first performed scRNA-seq of primary and metastatic gastric cancer samples, generating transcriptomes from nearly 43 000 individual cells. They then applied ML algorithms including unsupervised clustering (Seurat/Louvain), pseudotime TI (Monocle2), and Copy Number Variation analysis-based clonal inference (CopyKAT) to classify malignant and immune cells into distinct subclusters and reconstruct their evolutionary paths. This integration of single-cell profiling with computational modeling revealed four malignant epithelial programs (invasive/angiogenic, EMT, dormant, CSC-like) and organ-specific immune exhaustion signatures, thereby exposing the transcriptional heterogeneity that underlies metastatic behavior and patient prognosis.

## Disrupting cancer stem cell plasticity: functional screens, molecular mechanisms, and therapeutic implications

“CRISPR-based perturbation screens” with single-cell readouts have transformed our ability to study gene function and regulatory networks. These are large-scale experimental approaches in which systematic gene knockouts, knockdowns, or activations are introduced into cell populations to uncover how specific genes influence cellular behavior. By applying these perturbations across hundreds or thousands of genes in parallel, researchers can map genetic dependencies, regulatory circuits, and therapeutic vulnerabilities in a high-throughput manner. In particular, single-cell CRISPR screening (scCRISPR) technologies combine pooled CRISPR libraries with high-content phenotyping, enabling the dissection of complex cell states and interactions at single-cell resolution [92]. These platforms include scCRISPR coupled with RNA-seq, ATAC-seq, proteomics, or imaging, and they have advanced our understanding of genetic regulation, cancer biology, and therapy resistance.

An illustrative example is a recent “*in vivo* Perturb-seq” study that used CRISPRi with scRNA-seq to dissect tumor-intrinsic and microenvironmental drivers of glioblastoma, which is not a carcinoma but serves to illustrate how these approaches can be applied more broadly beyond epithelial cancers [93]. By delivering pooled sgRNA libraries into glioblastoma models and profiling responses with *in vivo* Perturb-seq, Liu *et al.* showed that loss of DNA damage repair genes sensitized tumors to radiotherapy, while perturbations of microenvironmental genes altered ligand-receptor signaling, cytokine secretion, and macrophage phagocytosis. These findings demonstrate that both tumor-intrinsic pathways and the surrounding microenvironment contribute to radiotherapy resistance. While such scCRISPR platforms uncover genetic vulnerabilities and therapy response regulators at high resolution, they rely on dissociated cells and therefore lose the spatial context of cell–cell interactions.

“Perturb-FISH”, developed by Binan *et al.*, addresses this limitation by combining pooled CRISPR interference with spatial transcriptomics in intact tumor sections, thereby preserving tissue architecture [94]. This approach enables researchers to measure both the direct effects of gene knockdowns and the indirect ripple-like consequences on neighboring cells within the tumor microenvironment. In melanoma xenografts, the study mapped how specific perturbations reshaped local immune states and inflammatory signaling. The ability to track how perturbations propagate through spatial niches offers a powerful framework for identifying regulators of tumor plasticity and potential CSC-supportive circuits in the future. Perturb-FISH thus sets a new benchmark for studying functional, spatially organized interactions between cancer cells and their surrounding stroma *in situ*.

Building on functional spatial perturbation approaches, observational spatial transcriptomics studies have further illuminated how CSC states are organized within tumor niches. A recent study by Arora *et al.* uncovered conserved transcriptional programs distinguishing the tumor core from the invasive leading edge in oral squamous cell carcinoma [95]. The leading edge was enriched in mesenchymal-like CSCs, while epithelial-like CSCs dominated the core. Importantly, the edge program correlated with poor prognosis and elevated EGFR signaling, underscoring how spatial CSC heterogeneity can influence tumor progression and therapeutic vulnerability.

Beyond individual readouts, recent advances increasingly integrate multiple single-cell omics with CRISPR perturbations and AI-based analysis. Combinations of scCRISPR with ATAC-seq, proteomics, or imaging link gene perturbations to regulatory, phenotypic, and spatial changes, while emerging single-cell DNA methylation techniques expand this framework to the epigenetic layer. For instance, a 2025 study introduced “Multiome Perturb-seq”, which extends traditional scCRISPR screens to simultaneously capture changes in gene expression and chromatin accessibility in response to each perturbation [96]. The study revealed that perturbations of chromatin remodelers induce distinct and sometimes uncoupled effects on transcription and accessibility, thereby uncovering regulatory programs that link epigenomic changes to transcriptional outcomes. This provides a clear example of multi-omics integration, linking transcriptional and epigenetic responses to gene perturbations within the same cells.

Together, these integrative approaches move beyond transcript-centric analyses and support a systems-level view of CSC plasticity and therapeutic resistance.

## Integrating multi-omics and artificial intelligence: toward a unified cancer stem cell atlas

**Figure 7:**
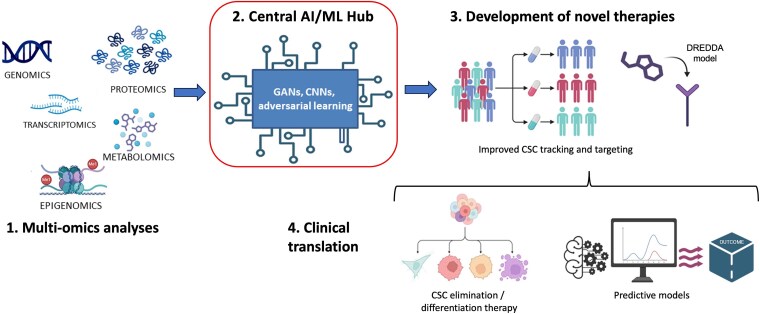
Future directions: integrating AI, multi-omics, and CSC research. Portray how ML and AI, combined with multi-omics data, could be shaping the next generation of CSC research and personalized oncology approaches. (i) Multi-omics including genomics, transcriptomics, proteomics, epigenomics, and metabolomics funneling into the AI engine. (ii) Central AI/ML Hub with deep-learning or machine-learning models (GANs, CNNs, adversarial learning) as the core. (iii) Outputs provide new therapeutic targets, drug repositioning (e.g. DREDDA model), improved classification and prognosis. (iv) Potential clinical benefits by establishing novel personalized medicine approaches, eventually resulting in improved outcome for cancer patients.

While scRNA-seq remains a foundational technology, integration with additional single-cell modalities enhances our ability to characterize CSC states. scATAC-seq adds chromatin accessibility profiles, helping to identify active transcriptional programs [97], while CITE-seq and spatial transcriptomics provide complementary protein expression and locational context (Fig. 7) [98]. The convergence of single-cell omics technologies and AI—with a focus on ML and its subset, DL—is revolutionizing biomedical research, particularly by advancing our understanding of cellular heterogeneity and cancer [99].

“Single-cell data are massive”, noisy, high-dimensional, and still sparse, posing critical analytical challenges that traditional methods struggle to address [100]. ML/DL algorithms are indispensable for extracting high-level features, identifying complex patterns, and building predictive models from these intricate datasets [101]. In single-cell omics, ML/DL can address data complexities like “dropout events” and “batch effects” through preprocessing (e.g. imputation, normalization) and already excels in downstream tasks such as cell type identification, TI, and multi-omics/spatial data integration [100, 102].

“scGDC” (single-cell Graph Diffusion Convolution) embeds cells in a denoised topological feature space via an auto-encoder with self-representation layer to learn a topological affinity graph, achieving superior clustering accuracy on 15 scRNA-seq datasets and retrieving elusive marker genes that conventional tools overlook [103]. Going one step further, “scLDS2” (single-cell Latent Discriminative Subspace 2) adopts an adversarial generative strategy to produce sparse yet biologically faithful synthetic cells, jointly optimizing sample generation, feature extraction, and clustering within a single end-to-end model; it significantly outperforms 17 state-of-the-art methods across 20 scRNA-seq datasets [104]. Together, scGDC and scLDS2 may offer complementary discriminative and generative strategies for identifying low-abundance CSCs or therapy-persisting clones, and could potentially be integrated into existing single-cell pipelines to help enrich the CSC census in heterogeneous tumors.

For CSC research, “ML/DL is starting to overcome long-standing challenges” in CSC identification, as CSCs often lack well-understood morphological features or unique and truly specific biomarkers [105]. DL models, including Convolutional Neural Networks (CNNs) and Conditional Generative Adversarial Networks, now enable automated, label-free morphological recognition of CSCs and prediction of CSC fate [106]. Recent advances have also introduced novel computational frameworks for integrating multimodal single-cell data.

For instance, the “Network-based Integration Clustering (NIC)” has been developed to jointly analyze scRNA-seq and epigenomic data for improved cell type identification [107]. NIC addresses the inherent sparsity and heterogeneity of single-cell data by adaptively learning cell similarity networks and extracting shared features through joint non-negative matrix factorization. Benchmarking across multiple datasets demonstrated that NIC significantly outperforms existing integration methods, enabling more accurate identification of cell types and extraction of biologically meaningful feature genes. Such integrative approaches hold promise for refining CSC classification by capturing both transcriptional and epigenetic dimensions of stemness, thereby contributing to a more holistic view of CSC identity and plasticity.

Moreover, “artificial intelligence (AI) accelerates the discovery of CSC biomarkers” and enables drug prioritization, identifying compounds that induce CSC differentiation, and offering promising new avenues for targeted and more efficient therapies [108].

“CytoTRACE2” is an interpretable DL framework that predicts both categorical potency states and absolute developmental potential from scRNA-seq data. Unlike earlier TI tools that yield dataset-specific relative orderings, CytoTRACE2 anchors predictions to six canonical potency categories (totipotent, pluripotent, multipotent, oligopotent, unipotent, differentiated), enabling cross-dataset comparability. Its core architecture is the Gene Set Binary Network, which learns compact, potency-associated gene programs by activating or deactivating individual genes, thus offering direct interpretability. The workflow converts single-cell transcriptomes into rank space, decodes potency-linked gene sets, computes enrichment scores per cell, and outputs both categorical labels and a continuous potency score (1 = totipotent, 0 = differentiated). The model was trained on 17 human and mouse scRNA-seq datasets (176 k cells across 18 tissues and 6 platforms) and tested on 13 independent datasets (363 k cells), achieving robust performance across species, tissues, and technologies. Its interpretable design also uncovered conserved gene programs of potency and revealed clinically relevant differentiation states in cancer [79].

Large language models (LLMs) are beginning to transform the field of single-cell omics by representing cells and biological information as “cell sentences”, allowing LLMs to “read” and “write” biological data. This approach, exemplified by Cell2Sentence-Scale (C2S-Scale), converts high-dimensional gene expression data into natural language, making complex single-cell data more accessible and interpretable. These transformer-based foundation models, like scGPT [109] and scBERT [110], are trained on vast datasets to learn gene–gene interactions and cellular patterns.

For instance, “C2S-Scale” can automatically generate biological summaries of scRNA-seq data and respond to complex biological questions in plain English, enabling conversational single-cell analysis [111]. Similarly, ChatNT interprets transcripts and proteins using natural language, further accelerating discovery and personalized medicine by allowing direct queries about cellular states or drug responses [112]. These strategies promise to move beyond marker-based CSC definitions and toward a unified, multiparameter CSC atlas, one that captures plasticity, context, and therapeutic relevance across different cancer types.

## Future perspectives: mapping and targeting cancer stem cell plasticity

The dynamic nature of CSCs remains a major challenge in oncology, particularly their capacity to evade current treatment approaches through reversible state changes. To address this, we propose the creation of a “cross-cancer CSC plasticity atlas”, a reference framework built from integrated single-cell, spatial, and functional datasets that captures key transitional states across cancers and treatment conditions.

Such an atlas would enable (i) the identification of lineage trajectories and high-entropy transition points as therapeutic bottlenecks, (ii) the rational design of state-specific interventions (e.g. blocking dedifferentiation, targeting metabolic dependencies in quiescent cells), and (iii) the prediction of therapy-induced plasticity routes to help overcome escape mechanisms of resistance. By decoding the logic of CSC state transitions and vulnerabilities, this approach could transform how we identify and eliminate the cellular roots of treatment failure.

However, it is important to acknowledge “current limitations” in this field. Pseudotime analysis has been instrumental in mapping the progression of cancer cells and identifying key transition points. For instance, a study on ovarian cancer utilized pseudotime trajectory analysis to uncover genes associated with cancer progression, offering potential targets for intervention [113]. However, the challenge lies in translating these gene associations into actionable therapeutic strategies. While pseudotime analysis offers valuable insights into the progression and plasticity of CSCs, translating these findings into effective therapies is an ongoing task. Targeting the dynamic and heterogeneous nature of CSCs requires a multifaceted approach, and current research is actively exploring these avenues. Continued studies are necessary to develop therapies that can effectively address the adaptability of CSCs identified through pseudotime analyses.

## Conclusion

CSC phenotypes are dynamic, driven by therapy-induced lineage plasticity, epigenetic reprogramming, and niche-specific cues rather than fixed markers or hierarchies. Recent single-cell and spatial advances—now including *in vivo* Perturb-seq [93] and multi-omics CRISPR screens such as Multiome Perturb-seq [96]–map how tumor-intrinsic programs and microenvironmental interactions co-evolve under treatment, identifying regulators of radio- and chemo-resistance and immune evasion with single-cell resolution. We therefore argue that targeting state transitions and circuit-level regulators (chromatin remodelers, EMT/metabolic switches, ligand-receptor axes) will be more impactful than static CSC depletion, especially when paired with spatially informed strategies that account for core versus invasive-edge programs [95, 114]. Looking ahead, integrating these causal, multimodal readouts with longitudinal sampling and computational modelling should accelerate translation into patient-stratified interventions.

While powerful, current approaches still face limitations—batch/technical variability, incomplete temporal sampling, and loss of spatial context in dissociated assays—now being narrowed by spatially resolved perturbation and integrated multi-omics that link chromatin accessibility to transcriptional outcomes in the same perturbed cells. Therefore, the field should prioritize (i) longitudinal, multi-omics single-cell designs to capture therapy-induced trajectories; (ii) spatially anchored models of CSC niches; and (iii) causal screens that bridge tumor and stromal compartments—directions supported by emerging work on treatment-induced stemness and lineage plasticity.

**Key Points**

- Single-cell technologies have transformed how cancer stem cell dynamics are studied.
- Integrative multi-omics and AI approaches reveal stemness as a dynamic, reversible state.
- Mapping tumor–microenvironment crosstalk enables therapeutic targeting of CSC plasticity.

## Data Availability

No new data were generated or analysed in support of this research. This review is based on previously published studies, all of which are cited in the reference list. The datasets discussed in this review are available in public repositories under the accession numbers provided.
